# The Deleterious Consequences of a Nonsteroidal Anti-inflammatory Drug in an Eisenmenger Patient

**DOI:** 10.1016/j.cjcpc.2023.10.011

**Published:** 2023-11-03

**Authors:** Sarah Grütter, David Haefliger, Grégoire Wuerzner, Patrick Yerly, Judith Bouchardy, Tobias Rutz

**Affiliations:** a Service of Cardiology, Lausanne University Hospital (CHUV) and University of Lausanne, Lausanne, Switzerland; b Division of Clinical Pharmacology, Lausanne University Hospital (CHUV) and University of Lausanne, Lausanne, Switzerland; c Division of Nephrology and Hypertension, Lausanne University Hospital (CHUV) and University of Lausanne, Lausanne, Switzerland

**We report a case of a patient with Eisenmenger’s syndrome who develops rapidly progressive dyspnea, desaturation, high blood pressure, and low platelet count after an ibuprofen intoxication of 4 g. The worsening oxygen saturation and elevated blood pressure were attributed to inhibition of the prostaglandin and prostacyclin synthesis, leading to systemic and pulmonary vasoconstriction followed by an increase of pulmonary vascular resistance, thus exacerbating the right-to-left shunt. The accompanying thrombocytopaenia was explained by an immune-mediated adverse reaction. After the clearance of ibuprofen, the patient’s oxygen saturation, blood pressure, and platelet count returned to the usual levels.**

## Case

We report on a 36-year-old patient known for Eisenmenger’s syndrome due to a nonrestrictive ventricular septal defect with a predominant right-left shunt associated with class I pulmonary arterial hypertension. The patient was under chronic macitentan (10 mg od) and sildenafil (20 mg tid) treatment. Because of neck pain due to muscular contraction for 1 week, she ingested a total of 9 g of paracetamol over 3 days (1 g every 8 hours) and 1 blister pack of 10 tablets of ibuprofen 400 mg (nonsteroidal anti-inflammatory drug, NSAID), that is, a total dose of 4 g over 2 days. She additionally took 300 mg of tolperisone (a muscle relaxant drug) once daily. As she subsequently developed over 2 days a rapidly progressive dyspnea, she presented to the emergency department of a local hospital.

On arrival at the emergency department, the patient presented with a high blood pressure of 169/110 mm Hg, accompanied by desaturation requiring an oxygen therapy of 15 L/min (FiO_2_ at 60%) to reach an oxygen saturation at rest of 80% (usual level at room air at 80%-83%). On physical examination, the patient was tachypneic (20 breaths/min), had a normal pulmonary auscultation, and showed no clinical signs of right or left heart failure. Because of the unstable clinical condition, the patient was transferred to the tertiary centre.

The blood gas analysis at arrival indicated hypoxemic respiratory failure without elevation of lactate. There were no clinical or laboratory signs for an infectious or inflammatory etiology. COVID-19 and influenza B + A polymerase chain reaction nasopharyngeal swab were negative. Laboratory tests revealed an increased N-terminal pro-B-type natriuretic peptide. Haemoglobin and platelet count were within usual ranges (Table 1). Computed tomography ruled out pulmonary embolism, oedema, and atelectasis. Further laboratory investigations eliminated common alternative causes of secondary hypertension including normal thyroid hormones, plasma metanephrines, and aldosterone.

**Table 1:** Clinical and laboratory parameters

Day	Systolic blood pressure (mm Hg)	Diastolic blood pressure (mm Hg)	O_2_ saturation—SaO_2_/SpO_2_ (%)	FiO_2_ (%)	Thrombocytes (G/L)	Creatinine (μmol/L)	Haemoglobin (G/L)	Nt-pro BNP (ng/L)
2 y	130	95	80	AA	113	71	222	90
−6 mo	117	90	80	AA	115	80	227	108
−6.5 mo			80	AA	112	86	221	
−3 mo	125	85	80	AA	83	86	227	
−2 mo			80	AA	114	80	217	
0	169	111	75	80	60	87	220	1015
1	159	111	67	100	–			
2	161	96	82	30	42		219	
3	153	99	83	35	37	65	216	
4	157	92	79	32	25		207	
5	150	86	82	28	22	62	212	
6	137	91	83	24	25	59	214	
7	129	100	83	28	38	62	224	
8	125	81	78	24	47		219	
9	129	89	80	AA	63		223	111
10	122	89	80	AA	85	79	217	

AA, ambient air; FiO_2_, fraction of inspired oxygen; NT-proBNP, N-terminal pro-B-type natriuretic peptide.

The patient was admitted to the intermediate care unit. A transthoracic echocardiography showed the previously known anatomy with a small left ventricle with normal systolic function, a dilated and hypertrophied right ventricle with normal systolic function, and the large nonrestrictive perimembranous ventricular septal defect with a right-left shunt.

The patient’s condition deteriorated rapidly, requiring a transfer to the intensive care unit for high-flow oxygen therapy for 24 hours (Table 1). In parallel, the platelet count decreased to a minimum value of 22 G/L (Table 1).

On the basis of the clinical picture of concomitant desaturation, arterial hypertension, and thrombocytopaenia, we concluded that NSAID toxicity was likely responsible for an increase of pulmonary vascular resistance (PVR) and worsening of the right-left shunt. We therefore increased the sildenafil dosage to 40 mg tid and maintained macitentan 10 mg od.

The patient’s condition improved over the following 9 days, with normalization of blood pressure, recovery of platelet count, and return of oxygen saturation to usual levels (Table 1).

## Discussion

We describe 3 adverse side effects of NSAIDs in a patient with Eisenmenger’s syndrome.

1. Arterial hypertension: prostaglandin E_2_ is a potent vasodilator and involved in the renal autoregulation of sodium and water.^1^ Through cyclooxygenase-2 inhibition, NSAIDs block the synthesis of prostaglandins (Fig. 1A).^2^ The reduced level of prostaglandin E_2_ results in systemic vasoconstriction and reduction of renal blood flow followed by sodium and water retention (Fig. 1A). In our case, the patient did not exhibit acute renal damage likely because of a preserved cardiac output maintaining the renal autoregulation. The systemic arterial hypertension was therefore probably caused by the systemic vasoconstriction.

**Figure 1:**
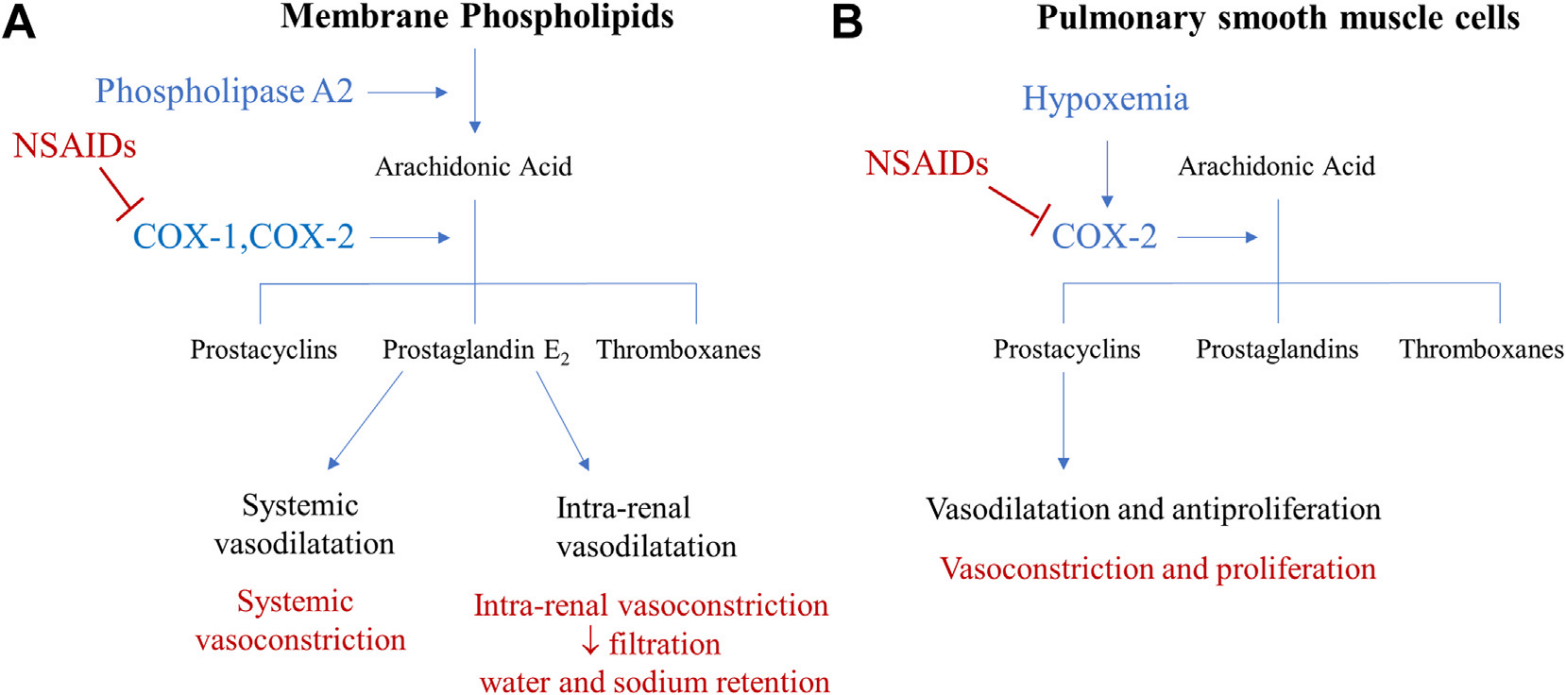
Mechanism of NSAIDs inducing high blood pressure (**A**) and exacerbation of pulmonary arterial hypertension (**B**). In red, interactions and consequences of NSAIDs with the prostaglandin and prostacyclin synthesis pathways. COX, cyclooxygenase; NSAID, nonsteroidal anti-inflammatory drug. Adapted from Burnier.^2^
2. Pulmonary arterial hypertension is characterized by abnormalities in the pulmonary endothelium resulting in disturbed syntheses of vasodilating and constrictive substances (nitric oxide, thromboxane A2, endothelin, and prostacyclin [PGI_2_]).^3^ PGI_2_ are primarily synthesized by vascular endothelial cells and potent vasodilators of the pulmonary arteries.^3^^,^^4^ NSAIDs reduce, by cyclooxygenase-2 inhibition, PGI_2_ levels in the pulmonary smooth muscle cells inducing pulmonary vasoconstriction and consequently an increase of PVR (Fig. 1B). One can speculate that the increased PVR led in our patient to an increase of the right-to-left shunt. However and unfortunately, transthoracic echocardiography did not allow us to evaluate changes of the degree of shunting due to a reduced image quality, and we refrained from insertion of a Swan-Ganz catheter due to the inherent risk of such catheters in patients with a right-to-left shunt.
3. Our patient had chronic thrombocytopaenia that is typically found in Eisenmenger’s syndrome.^5^ Common etiologies for the decline in platelet count were ruled out. We excluded heparin-induced thrombocytopaenia, thrombosis, disseminated intravascular coagulation, infectious disease (cytomegalovirus, Epstein-Barr virus, HIV, viral hepatitis, and *Helicobacter pylori*), hypersplenism, folic acid and B12 deficiency, and autoimmune etiology. Drug-induced aggravated thrombocytopaenia was therefore suspected. Initially, sertraline and macitentan were incriminated but finally excluded as the cause as the platelet count returned to its usual level despite their continuation throughout the hospitalization. Therefore, ibuprofen remains the only suspected drug. Thrombocytopaenia is a known adverse effect of ibuprofen. A case report describes a patient who received a total of 2.6 g of ibuprofen over 6 days who developed on the sixth day thrombocytopaenia of 6 G/L, which recovered after discontinuation of the treatment and administration of prednisolone and immunoglobulins.^6^ A further report describes a 71-year-old patient who developed thrombocytopaenia after the administration of 2.4 g of ibuprofen who also recovered after prednisolone and immunoglobulin administration.^7^ In both cases, an immune-mediated reaction was suspected with antibody-mediated platelet destruction. Consistent with these reports, we observed an improvement of the platelet count in our patient 7 days after stopping ibuprofen, however, without the need of corticoid or immunoglobulin administration.

## Conclusions

• Like in the general population, nonsteroidal anti-inflammatory drugs (NSAIDs) are frequently prescribed as first-line medication for acute pain in patients with pulmonary arterial hypertension.
• Administration of NSAIDs can have potentially deleterious consequences in these patients, particularly on pulmonary vascular resistance, platelet count, and systemic arterial pressure.
• Consequently, prescription of NSAIDs should be avoided in patients with pulmonary arterial hypertension similar to other patient populations with cardiovascular disease.
